# APOBEC3 Deletion is Associated with Breast Cancer Risk in a Sample of Southeast Iranian Population

**Published:** 2015

**Authors:** Maryam Rezaei, Mohammad Hashemi, Seyed Mehdi Hashemi, Mohammad Ali Mashhadi, Mohsen Taheri

**Affiliations:** 1 *1.Cellular and Molecular Research Center, Zahedan University of Medical Sciences, Zahedan, Iran.*; 2 *Department of Clinical Biochemistry, School of Medicine, Zahedan University of Medical Sciences, Zahedan, Iran.*; 3 *Department of Internal Medicine, School of Medicine, Zahedan University of Medical Sciences, Zahedan, Iran.*; 4 *4.Genetics of Non-Communicable Diseases Research Center, Zahedan University of Medical Sciences, Zahedan, Iran.*

**Keywords:** *APOBEC3*, breast Cancer, polymorphism, deletion

## Abstract

Breast Cancer (BC) is considered as one of the most important causes of death worldwide. Previous studies showed that apolipoprotein B mRNA- editing catalytic polypeptide-like 3 (APOBEC3) gene deletion significantly increased the risk of BC risk in Chinese and European women. The present study aimed to assess the possible impact of *APOBEC3* deletion and the risk of BC in a sample of Iranian population. The *APOBEC3* insertion/deletion (I/D) was analyzed in a case- control study including 262 BC patients and 217 healthy women. Polymerase chain reaction (PCR) was used to genotype the variant in *APOBEC3* gene. The findings of this study showed that I/D as well as I/D+D/D genotype increased the risk of BC (OR= 1.57, 95% CI= 1.07- 2.31, p= 0.025 and OR= 1.50, 95% CI= 1.03- 2.19, p= 0.037, respectively) in comparison with I/I genotype. In conclusion, our findings suggest that *APOBEC3* deletion polymorphism increased the risk of BC in an Iranian population in the southeast of Iran.

Every day many women are diagnosed with breast cancer (BC) all over the world. It can be considered the most common cancer amongst females globally and is also the leading cause of cancer-related death in many countries (1). BC is recognized as an important health care problem worldwide affecting approximately 1 million women annually (1-3). BC is also reported as one of the most frequent malignancies among Iranian women, comprising 21.4% of female cancers in this population (4). Interestingly, it was reported that BC affects Iranian women about a decade earlier than Western countries (5) which highlights the importance of research on BC in Iranian population. Several different factors are involved in BC pathogenesis while its exact etiology is complicated and not clearly identified. Our recent publications have provided evidence that genetic factors play important roles in the pathogenesis and progre-ssions of BC in the population of southeast of Iran (6-10).

Somatic mutations in the genome are critical for transformation of normal cells into cancers. The existence of hundreds to thousands of mutations has been shown in most cancers (11-14).

*APOBEC3s* (A3s) gene cluster is located on chromosome 22q13.1-q13.2 including *APOBEC3A*, *APOBEC3B*, *APOBEC3C*, *APOBEC3D*, *APOBE-C3E*, *APOBEC3F*, *APOBEC3G*, and *APOBEC3H* which are arranged in tandem and play key roles in intracellular defense against viral infection (15, 16). They all have the cytidine deaminase activity and are able to convert cytosine to uracil (15).

Through copy number variation (CNV) genome wide association study (GWAS), Long et al. realized a common (CNV) locus for breast cancer in Chinese women, which was located between the fifth exon of *APOBEC3A* and the eighth exon of *APOBEC3B *(17). The finding was also repeated among women of European ancestry (18). It has been shown that a 29.5 Kb deletion occurs between the fifth exon of *APOBEC3A *and the eighth exon of *APOBEC3B*, leading to the complete removal of the *APOBEC3B *coding region. Several studies have found an association between *APOBEC3 *deletion and risk of various cancers (17-19).

To the best of our knowledge there is no any report regarding the association between *APOBEC3* deletion and BC in Iranian women, therefore in the present study we aimed to find out the impact of *APOBEC3* deletion on BC risk in a sample of Iranian population.

## Material and methods

**Patients**

This population- based case- control study was accomplished on 262 pathologically confirmed BC patients referred to Ali Ebneh Abitaleb hospital (Iran) from February 2009 until August 21014 and 217 age-matched population-based healthy women with no history of any type of cancer and not related to the patients. Ethical approvals for recruitment were obtained from the local Ethics Committee of Zahedan University of Medical Sciences, and informed consent was obtained from all patients and healthy individuals. Blood samples were collected in EDTA-containing tubes from patients and healthy controls and DNA was extracted using the salting out method. The quality of the isolated DNA was checked by electrophoresis on 1% agarose gel, quantitated spectrophotometrically and stored at -20 °C till further use.

***APOBEC3B*** **insertion/deletion genotyping**

Genotyping of *APOBEC3 *insertion/deletion was performed using polymerase chain reaction (PCR) as described by Kidd et al. (20). One pair of deletion and two pairs of insertion (insertions 1 and 2) primers were used to distinguish between the insertion and deletion alleles. The primers sequences used were: Deletion-F: 5`-TAGGTGC-CACCCCGAT-3`, Deletion-R: 5`-TTGAGCAT-AATCTTACTCTTGTAC-3`, Insertion1-F: 5`-TTGGTGCTGCCCCCTC-3`, Insertion1-R: 5`-TAGAGACTGAGGCCCAT-3`, Insertion2-F: 5`-TGTCCCTTTTCAGAGTTTGAGTA-3`, Insertion-2-R: 5`-TGGAGCCAATTAATCACTTCAT-3`. Deletion primers are specific to the deletion sequence configuration and generate a 700 bp PCR product upon amplification. Insertion 1 and Insertion 2 primers amplify only the insertion configuration and produce 490 and 705 bp products, respectively. Insertion and deletion PCR assays were performed separately, the products pooled, and visualized on a standard 2% agarose gel. In addition, each of the samples, which appeared to be homozygous for the deletion, was genotyped using a second set of oligonucleotides for the insertion (Insertion 2).

**Fig. 1:**
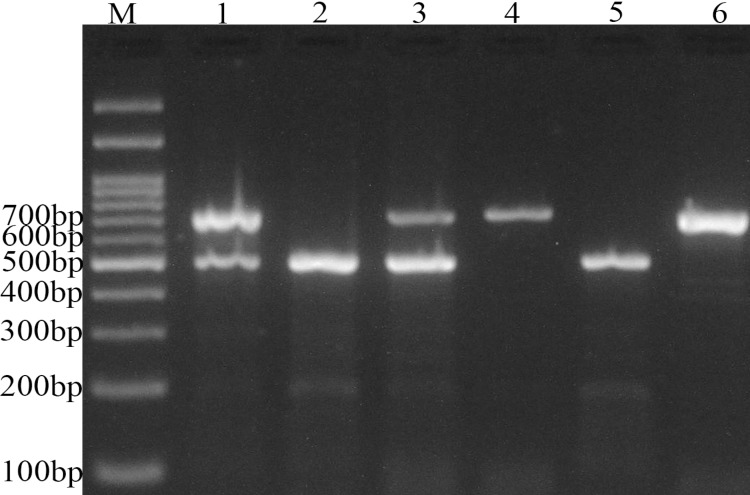
Photograph of DNA electophoresis for detection of *APOBEC3 *I/D variant. M: DNA marker; lanes 1, and 3: I/D; lane 2, and  5: D/D; lanes 4 and 6, I/I genotypes

PCR was performed using 2X Prime Taq Premix (Genet Bio, Korea). The amplification procedure consisted of an initial denaturing step for 5 min at 95 °C followed by 34 cycles: for 30 s at 95 °C, 24 s at 61 °C, and 17 s at 72 °C, as well as a final extension step for 10 min at 72 °C. The PCR products were visualized on a 3% agarose gel containing 0.5 µg/ ml of ethidium bromide (Figure 1).

**Statistical analysis**

All statistical analyzes were computed using statistical package SPSS 20 software. The differences between the variables were evaluated by chi-square test or independent sample t-test according to the data. The association between genotypes and BC were assessed by computing the odds ratio (OR) and 95% confidence intervals (95% CI) from logistic regression analyses. A p- value < 0.05 was considered as statistically significant. According to our results, sample power was computed for *APOBEC3* deletion by comparison of each genotype with the sum of other genotypes by using STATA 10 software (Table 1).

**Table 1:** Association between the *APOBEC3* gene deletion and breast cancer risk

**APOBEC3 I/D variant**	**Cases** **n (%)**	**Controls** **n (%)**	**OR (95% CI)**	**P- value**	**Study power%**
I/I	154 (58.8)	148 (68.2)	1.00	-	53
I/D	103 (39.3)	63 (29.0)	1.57 (1.07-2.31)	0.025	62
D/D	5 (1.9)	6 (2.8)	0.80 (0.24-2.66)	0.767	6
I/D+D/D	108 (41.2)	69 (31.8)	1.50 (1.03-2.19)	0.037	53
Allele					
I	411 (78.4)	359 (82.7)	1.00	-	35
D	113 (21.6)	75 (17.3)	1.32 (0.95-1.82)	0.102	35

**Table 2:** Association between *APOBEC3A* I/D polymorphism and clinicopathological characteristics

**Variables**	***APOBEC3A***			***P***
	I/I	I/D	D/D	
Age (years)				0.696
≤50	84	60	2	
>50	64	42	3	
Pathological type				0.138
Ductal	94	74	5	
Others	47	25	0	
Tumor size (cm)				0.429
≤2	35	47	1	
>2	77	79	7	
TNM Stage				0.850
I	19	22	3	
II	47	48	5	
III	32	35	3	
IV	13	24	2	
Grade				0.437
I	23	21	2	
II	64	67	7	
III	14	30	1	
IV	0	0	0	
ER status				0.858
Positive	68	78	8	
Negative	37	43	3	
PgR status				0.627
Positive	62	79	6	
Negative	41	42	5	
HER2 status				0.155
Positive	50	69	9	
Negative	61	59	4	

## Results

The study groups included 262 pathologically confirmed BC patients with an average age of 48.9± 11.2 years and 217 healthy women with a mean age of 50.0± 13.0 years. The demographic information of patients group was summarized in Table 1. No significant difference was found between the groups regarding age (p= 0.306). The frequency distribution of the *APOBEC3* I/D in BC cases and controls are shown in Table 1. Our results showed that I/D as well as I/D+D/D genotype increased the risk of BC (OR= 1.57, 95% CI= 1.07-2.31, p= 0.025 and OR= 1.50, 95% CI= 1.03-2.19, p= 0.037, respectively) in comparison with I/I genotype.

The D allele frequency in BC and controls were 0.216 and 0.173, respectively. The *APOBEC3* D allele was not associated with the risk of BC in comparison with I allele (OR= 1.32, 95% CI= 0.95-1.82, p= 0.102). 

The *APOBEC3 *I/D in controls (χ2= 0.052, p= 0.819) but not in cases (χ2= 6.88, p= 0.008) were in Hardy-Weinberg Equilibrium (HWE).

As shown in table 2, no significant association was observed among *APOBEC3 *deletion and clinicopathological parameters, including tumor stage, tumor grade, estrogen and progesterone receptors (ER, PgR), tumor size, and human growth factor receptor 2 (HER2).

## Discussion

In the current study we examined the impact of *APOBEC3* deletion on BC risk in a sample of Iranian population who were living in southeast of this country. The findings revealed that *APOBEC3* deletion significantly increased the risk of breast cancer among a sample of Iranian women. Long et al. performed a genome-wide

association study in Chinese population and found a strong association between common deletion in the *APOBEC3* gene and BC (17). Their findings showed the ORs of 1.31 (95% CI= 1.21-1.42) for a one-copy deletion and 1.76 (95% CI= 1.57-1.97) for a two-copy deletion. The result was also replicated among women of European ancestry and the findings showed that the deletion was significantly associated with breast cancer risk, with ORs and 95% CIs of 1.21 (1.02–1.43) for one-copy deletion and 2.29 (1.04–5.06) for two-copy deletion in comparison with no deletion (18).

Qi et al. investigated the possible association between *APOBEC3* gene deletion and risk of epithelial ovarian cancer (EOC) in Chinese population (19). They found that one copy deletion as well as two-copy deletion of *APOBEC3* significantly increased the risk of EOC in comparison with subjects with no deletions.

Addition evidences suggested that *APOBEC3B* is involved in mutagenesis of human cancers such as bladder, cervix, lung (adenocarcinoma and squamous cell carcinoma), head and neck as well as BC (21-24). *APOBEC3B* catalyzes genomic DNA deamination and it is a main mutational source in BC accounting for C-to-T transitions (25). Upregulation of *APOBEC3B* has been revealed in BC showing that it as an important source of enzymatic mutation and DNA damage that could inactivate the tumor suppressor *TP53* gene (25).

We found no significant association between *APOBEC3* deletion and clinicopathological parameters of BC patients. To the best of our knowledge, there is no data regarding the impact of *APOBEC3 *deletion on response to treatment as well as prognosis of BC patients.

In conclusion, our findings indicated that *APOBEC3* deletion significantly increased the risk of BC in an Iranian population in southeast of Iran. Large sample sizes in diverse ethnicities are still required to certify our finding.
